# HIV-1 viral protein R (Vpr) induces fatty liver in mice via LXRα and PPARα dysregulation: implications for HIV-specific pathogenesis of NAFLD

**DOI:** 10.1038/s41598-017-13835-w

**Published:** 2017-10-17

**Authors:** Neeti Agarwal, Dinakar Iyer, Chiara Gabbi, Pradip Saha, Sanjeet G. Patel, Qianxing Mo, Benny Chang, Biman Goswami, Ulrich Schubert, Jeffrey B. Kopp, Dorothy E. Lewis, Ashok Balasubramanyam

**Affiliations:** 1 Translational Metabolism Unit, Diabetes Research Center, Division of Diabetes, Endocrinology and Metabolism, Department of Medicine, Baylor College of Medicine, Houston, TX USA; 2 University of Houston, Department of Biology and Biochemistry, Houston, TX USA; 3 Department of Surgery, UCLA, Los Angeles, CA USA; 4 Dan L. Duncan Cancer Center, Baylor College of Medicine, Houston, TX USA; 5 Institute of Virology, Friedrich-Alexander University Erlangen-Nürnberg (FAU), Erlangen, Germany; 6 Kidney Disease Section, National Institute of Diabetes and Digestive and Kidney Diseases, National Institutes of Health, Bethesda, MD USA; 7 Division of Infectious Diseases, Department of Medicine, University of Texas Health Sciences Center, Houston, TX USA; 8 Endocrine Service, Ben Taub General Hospital, Houston, TX USA; 9 Present Address: Department of Molecular Biology, University of Houston, Houston, TX USA

## Abstract

HIV patients develop hepatic steatosis. We investigated hepatic steatosis in transgenic mice expressing the HIV-1 accessory protein Vpr (Vpr-Tg) in liver and adipose tissues, and WT mice infused with synthetic Vpr. Vpr-Tg mice developed increased liver triglyceride content and elevated ALT, bilirubin and alkaline phosphatase due to three hepatic defects: 1.6-fold accelerated *de novo* lipogenesis (DNL), 45% slower fatty acid ß-oxidation, and 40% decreased VLDL-triglyceride export. Accelerated hepatic DNL was due to coactivation by Vpr of liver X receptor-α (LXRα) with increased expression of its lipogenic targets *Srebp1c, Chrebp, Lpk*, *Dgat*, *Fasn* and *Scd1*, and intranuclear SREBP1c and ChREBP. Vpr enhanced association of LXRα with *Lxrα* and *Srebp1c* promoters, increased LXRE-LXRα binding, and broadly altered hepatic expression of LXRα-regulated lipid metabolic genes. Diminished hepatic fatty acid ß-oxidation was associated with decreased mRNA expression of *Pparα* and its targets *Cpt1*, *Aox*, *Lcad*, *Ehhadh*, *Hsd10* and *Acaa2*, and blunted VLDL export with decreased expression of *Mttp* and its product microsomal triglyceride transfer protein. With our previous findings that Vpr circulates in HIV patients (including those with undetectable plasma HIV-1 RNA), co-regulates the glucocorticoid receptor and PPARγ and transduces hepatocytes, these data indicate a potential role for Vpr in HIV-associated fatty liver disease.

## Introduction

The high prevalence of hepatic steatosis in HIV-1-positive patients^1–3^ is commonly ascribed to coinfection with hepatitis C virus (HCV) or adverse effects of antiretroviral (ART) drugs, especially dideoxynucleoside analogues^4,5^. However, recent papers indicate that 30–70% of HIV-positive patients have non-alcoholic fatty liver disease (NAFLD) without hepatitis C co-infection^2–4,6–8^. While these reports do not distinguish the effects of ART from those of the virus *per se* on the development of hepatic steatosis, they suggest that a factor intrinsic to HIV-1 could play an etiologic role. Identifying mechanisms whereby HIV-1 causes NAFLD would provide novel insights into a viral etiology of this complex condition.

Viral protein R (Vpr), an HIV-1 accessory protein, permits efficient virion assembly, nuclear translocation of the preintegration complex, nucleocytoplasmic shuttling, apoptosis and transcription of the HIV-1 long terminal repeat and host cell genes^9^. Vpr is produced by HIV-1 even following inhibition of viral replication by protease inhibitors^10^, can transduce cells in a receptor- and energy-independent manner, and localize in the nucleus and mitochondria^11^. We demonstrated in two mouse models (one expressing the Vpr transgene in liver and adipose tissue, the other with chronic infusion of synthetic Vpr) that this protein is a promiscuous coregulator of transcription factors for host genes that modulate energy metabolism, e.g., coactivating the glucocorticoid receptor (GR) and corepressing peroxisome proliferator activated receptor-γ (PPARγ) and PPARα^12^. These transcriptional effects cause lipid kinetic, biochemical and histologic abnormalities that recapitulate HIV-associated lipodystrophy syndrome^13^. We also showed that virion-free Vpr in plasma is sufficient to cause these effects because it transduces adipocytes and hepatocytes in the absence of intact HIV-1 and persists in the circulation of HIV patients receiving “viral-suppressive” ART^12^.

Our previous studies were focused on the effects of Vpr on adipose function, but we observed that both mouse models also develop hepatic steatosis without high fat consumption^12^. Increased hepatic fatty acid flux from accelerated lipolysis, as we previously reported, could contribute to this phenotype. In the present study, we investigated comprehensively the mechanisms whereby Vpr induces fatty liver in these mice. We found that Vpr transgenic (Vpr-Tg) mice exhibit the following hepatic defects: increased *de novo* lipogenesis (DNL), decreased fatty acid oxidation and blunted very low density lipoprotein-triglyceride (VLDL-TG) export. The molecular mechanisms extend beyond the previously demonstrated Vpr-mediated coregulation of GR, PPARγ and PPARα to include coactivation of liver X receptor-α (LXRα), a master regulator of DNL. In conjunction with our earlier demonstration that Vpr circulates in the blood of HIV patients on ART, including those without detectable plasma viral load, they support the concept that Vpr may be a causal agent in the development of a unique form of NAFLD.

## Results

### Vpr mice have elevated liver enzymes and plasma triglyceride levels

Compared to WT mice, Vpr-Tg mice had elevated fasting plasma triglycerides (120 ± 13 vs. 88 ± 6 mg/dL, P = 0.03), AST (137 ± 11 vs. 96 ± 14 IU/L, P = 0.03), alkaline phosphatase (40 ± 5 vs. 27 ± 3 IU/L, P = 0.03) and total bilirubin (2.1 ± 0.3 vs. 1.1 ± 0.13 mg/dL, P = 0.01) levels (Table 1). sVpr-treated compared to vehicle-treated mice had elevated fasting plasma triglycerides (71.5 ± 6.65 vs. 44.3 ± 1.86 mg/dL, P = 0.005), alkaline phosphatase (9.6 ± 1.4 vs. 5.35 ± 0.12 IU/L, P = 0.02) and total bilirubin (0.17 ± 0.03 vs. 0.06 ± 0.02 mg/dL, P = 0.01) levels (Table 2).

**Table 1:** Plasma triglycerides and liver functions in Vpr-Tg and WT mice.

	WT ± SE (n = 10)	Vpr±SE (n = 14)	P
Triglyceride (mg/dL)	87.56 ± 6.04	120.05 ± 13.21	0.03
ALT (IU/L)	47.40 ± 6.87	67.85 ± 9.99	0.12
AST (IU/L)	96.40 ± 13.6	136.56 ± 11.2	0.03
Alk Phos (IU/L)	26.58 ± 2.67	40.38 ± 5.05	0.03
Total Bilirubin (mg/dL)	1.09 ± 0.13	2.11 ± 0.29	0.01

**Table 2:** Plasma triglycerides and liver functions in sVpr- and vehicle-treated mice.

	Vehicle ± SE (n = 7)	sVpr ± SE (n = 7)	P
Triglyceride (mg/dL)	44.3 ± 1.86	71.5 ± 6.65	0.005
ALT (IU/L)	72.05 ± 4.46	83.4 ± 11.3	0.379
AST (IU/L)	134.19 ± 17.46	190.13 ± 47.03	0.298
Alk Phos (IU/L)	5.35 ± 0.12	9.6 ± 1.4	0.02
Total Bilirubin (mg/dL)	0.06 ± 0.02	0.17 ± 0.03	0.01

### Liver fat is increased in Vpr mice

**Figure 1:**
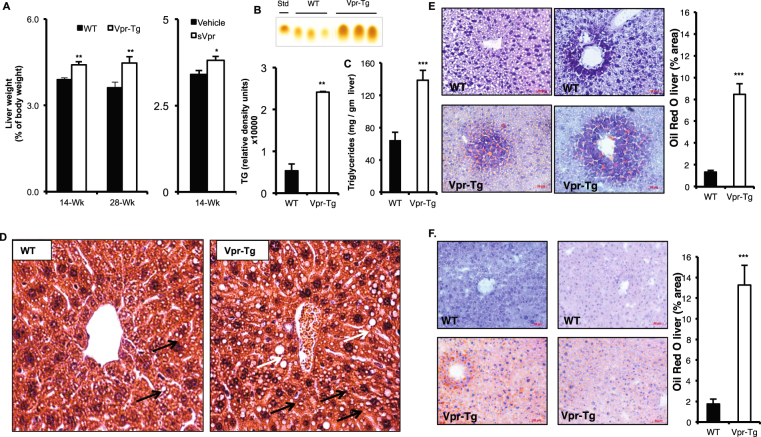
Vpr transgenic mice develop hepatosteatosis. (**A**) Increased liver mass (normalized to body weight) was present in Vpr-Tg compared to WT mice of 14 week old mice (n = 5–6 per group) and 28 week old mice (n = 4–5 per group) and sVpr- compared to vehicle-treated mice (n = 7 per group). (**B**) Increased liver triglyceride content was present in Vpr-Tg mice by TLC (n = 3 per group) and (**C**) by colorimetric assay (n = 5–6 per group). (**D**) Steatosis was observed in Vpr-Tg liver (hematoxylin-eosin stain). Left panel shows liver parenchyma around a centrilobular vein in WT mouse, with a pattern of microsteatosis (black arrows). Right panel shows peri-centrilobular area of liver in Vpr-Tg mouse, with both microsteatosis (black arrows) and macrosteatosis (white arrows). Scale bar = 50 μm. (**E**) Oil Red O–stained liver sections of 14-week Vpr-Tg show increased lipid accumulation compared to WT mice. Bar graph shows quantification of ORO-stained area. (**F**) Oil Red O–stained liver sections of 28-week Vpr-Tg show progressive lipid accumulation compared to WT mice. Bar graph shows quantification of ORO-stained area. Values are mean ± SE. *P < 0.05, **P < 0.01, ***P < 0.001.

Liver weight as a percent of body weight was increased in both 14 week-old (4.41 ± 0.20 vs. 3.89 ± 0.07%, P < 0.05) and 28 week-old (4.47 ± 0.21 vs. 3.61 ± 0.11%, P < 0.05) Vpr-Tg mice compared to WT littermates. 14-week old sVpr-treated mice also had increased liver weight as a percent of body weight compared to vehicle-treated mice (3.8 ± 0.10 vs. 3.4 ± 0.10, P = 0.02) (Fig. 1A). Hepatic triglyceride content was increased in liver of Vpr-Tg compared to WT mice, both as measured by TLC (24148 ± 187 vs. 5350 ± 1649 relative density units, P < 0.01) and by a colorimetric method (138.7 ± 12.4 vs. 64 ± 9.8 mg/g liver, P < 0.001) (Fig. 1B,C). Livers from Vpr-Tg mice demonstrated a pattern of both microvesicular and macrovesicular steatosis in ~40% of the hepatocytes, mainly with a pan-acinar distribution, whereas liver of WT mice showed a mild microvesicular steatosis in ~25% of the hepatocytes (Fig. 1D). Oil Red O stained area was increased in 14 week-old Vpr-Tg compared to WT liver (8.46 ± 0.96 vs. 1.32 ± 0.18%, P < 0.001) (Fig. 1E) and 28 week-old Vpr-Tg compared to WT liver (13.27 ± 1.9 vs. 1.75 ± 0.47%, P < 0.001) (Fig. 1F). Liver fat content as assessed by Oil Red O staining increased significantly as the Vpr-Tg mice aged (P < 0.05), with no significant change over time for the WT mice.

### DNL is increased, and expression of SREBP1c and ChREBP-regulated genes and of the corresponding DNL enzymes is upregulated, in liver of Vpr mice

**Figure 2:**
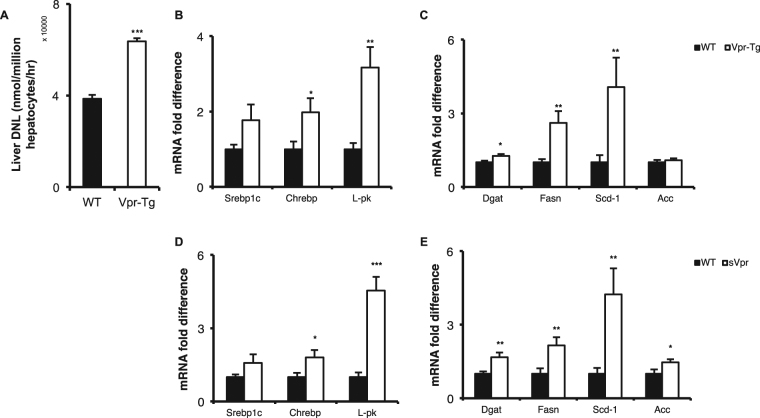
Increased *de novo* lipogenesis and mRNA levels of LXR-regulated lipogenic genes in liver of Vpr-Tg and sVpr-treated mice. (**A**) Faster conversion of [^14^C]acetate into [^14^C]-labeled fatty acids in primary hepatocytes was noted in Vpr-Tg compared to WT mice (N = 3 per group). (**B**) Increased mRNA levels of *Chrebp* and its target *Lpk* were present in Vpr-Tg compared to WT mice (N = 8 per group). (**C**) mRNA levels of *Dgat, Fasn and Scd1* were present in Vpr-Tg compared to WT mice (N = 8 per group). (**D**) mRNA levels of *Chrebp* and *Lpk* were increased, and there was a trend towards increased mRNA level of *Srebp1c* (P = 0.077), in sVpr-treated compared to vehicle-treated mice (N = 8 per group). (**E**) Increased mRNA levels of *Dgat, Fasn, Scd1 and Acc* were noted in sVpr-treated compared to vehicle-treated mice (N = 8 per group). Values are mean ± SE. *P < 0.05, **P < 0.01, ***P < 0.001.

Fatty acid synthesis (DNL) rate was faster in primary hepatocytes of Vpr-Tg compared to WT mice (63634.17 ± 1280.89 vs. 38537.98 ± 1723.45 nmoles/10^6^ hepatocytes/h, P < 0.05) (Fig. 2A). Since DNL is regulated by the amount and activity of the master transcription factors sterol regulatory element binding protein-1c (SREBP1c) and carbohydrate regulatory element binding protein (ChREBP)^14,15^, we quantified mRNA expression of both *Chrebp* and *Srebp1c* and of their key target genes in liver of Vpr-Tg compared to WT mice and of sVpr-treated compared to vehicle-treated mice. mRNA levels of *Chrebp* and the canonical ChREBP target gene liver pyruvate kinase (*Lpk* **)** were increased in both Vpr mouse models compared to their respective control mice; the mRNA level of *Srebp1c* was not significantly increased in Vpr-Tg compared to WT mice but showed a trend (P = 0.077) towards increase in sVpr-treated compared to vehicle-treated mice (Fig. 2B,D). The mRNA levels of diglyceride acyltransferase (*Dgat*), fatty acid synthase (*Fasn*), and stearoyl coenzyme A desaturase (*Scd-1*), critical lipogenic gene targets of SREBP1c and ChREBP, were significantly upregulated in Vpr-Tg compared to WT mice, whereas that of acetyl co-A carboxylase (*Acc*) was not different (Fig. 2C). In liver of sVpr-treated compared to vehicle-treated mice, there were significant increases in *Dgat*, *Fasn*, *Scd*-1 and *Acc* mRNA levels (Fig. 2E).

**Figure 3:**
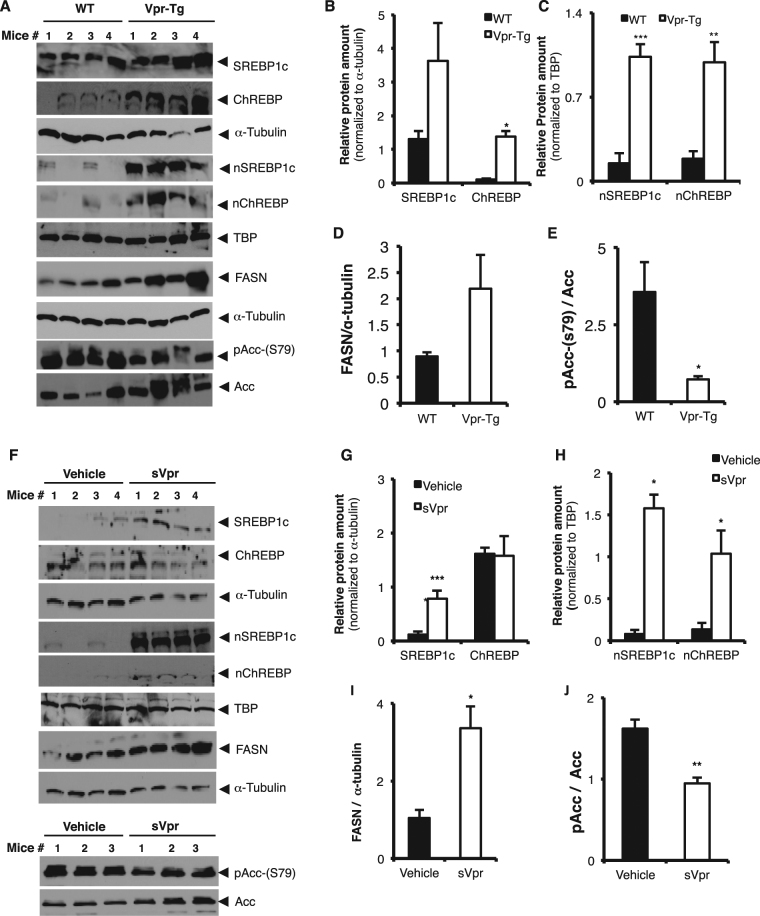
Increased amounts and activation of regulatory lipogenic proteins in liver of Vpr-Tg and sVpr-treated mice. (**A**) Immunoblots show expression of transcription factors and their target proteins in Vpr-Tg vs. WT mice (N = 4 per group). (**B**) There was a trend towards increased SREBP1c (P = 0.1) and increased ChREBP expression in whole cell extracts of Vpr-Tg compared to WT mice (N = 4 per group). (**C**) SREBP1c and ChREBP amounts were increased in nuclear fractions of Vpr-Tg compared to WT mice (N = 4 per group). (**D**) There was a trend towards increased FASN (p = 0.1) in Vpr-Tg compared to WT mice (N = 4 per group). (**E**) The ratio of phosphoS79-Acc to ACC was decreased in whole cell extracts of Vpr-Tg compared to WT mice (N = 4 per group). (**F**) Immunoblots show expression of transcription factor and their target proteins in sVpr-treated vs. vehicle-treated mice (N = 4 per group; N = 3 per group for ACC/pACC). (**G**) There was increased SREBP1c but not ChREBP in whole cell extracts of sVpr-treated compared to vehicle-treated mice (N = 4 per group). (**H**) There was increased SREBP1c and ChREBP in nuclear fractions of sVpr-treated compared to vehicle-treated mice (N = 4 per group). (**I,J**) There were increased FASN (N = 4 per group) and decreased ratio of phosphoS79-ACC to ACC (N = 3 per group) in whole cell extracts of sVpr-treated compared to vehicle-treated mice. Multiple replicate immunoblots were prepared for each experiment with 50 µg total protein loaded per lane, and each immunoblot probed was with a different antibody.Values are mean ± SE. *P < 0.05, **P < 0.01. [Note: Immunoblot figures have been cropped to show relevant bands. Original uncropped blots are shown in Supplementary Fig. S2A and B. No image enhancement was used, but the bluish background of the original autoradiogram was changed to gray].

In parallel with these differences in mRNA expression, total protein levels of ChREBP were significantly increased in liver of Vpr-Tg compared to WT mice and SREBP1c showed a trend (P = 0.1) towards increase in Vpr-Tg compared to WT mice (Fig. 3A,B). In liver of sVpr-treated mice, total SREBP1c was increased compared to vehicle-treated mice but total ChREBP was not (Fig. 3F,G). Intranuclear protein levels of both SREBP1c and ChREBP were significantly increased in Vpr-Tg compared to WT mice (Fig. 3A,C); similarly, intranuclear levels of both SREBP1c and ChREBP were significantly increased in liver of sVpr-treated mice compared to vehicle-treated mice (Fig. 3F,H). Protein expression of FASN showed a trend (P = 0.1) towards increase in Vpr-Tg compared to WT mice (Fig. 3A,D) and was significantly increased in sVpr-treated compared to vehicle-treated mice (Fig. 3F,I). The ratio of phosphorylated ACC-phospho-S79 to ACC (Fig. 3A,E) was decreased significantly, indicating shunting away from (AMP kinase-activated) fat oxidation towards fatty acid synthesis, in Vpr-Tg compared to WT mice. Similar results were observed in sVpr-treated compared to vehicle-treated mice (Fig. 3F,J).

### Vpr binds to LXRα and enhances LXRα-regulated promoter activity

**Figure 4:**
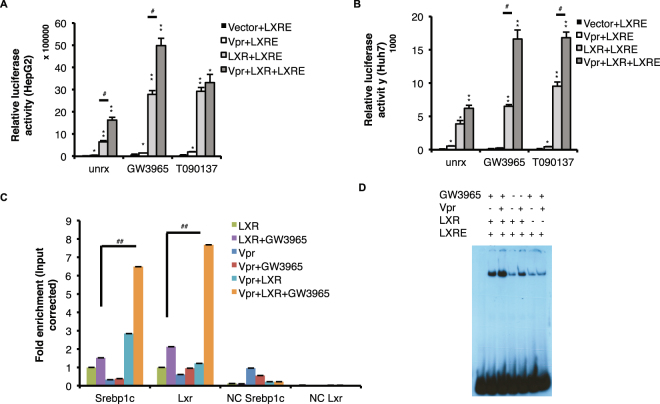
Vpr binds to LXRα and enhances LXRE-dependent promoter activity. (**A**) Vpr increased luciferase activity in HepG2 cells transfected with a LXRE-luciferase construct, with or without exogenous LXRα, in the presence or absence of LXR agonists GW3965 or T090137. (**B**) Vpr increased luciferase activity in Huh7 cells transfected with an LXRE-luciferase construct, with or without exogenous LXRα, in the presence or absence of LXR agonists GW3965 or T0901317. (**C**) Vpr increased association of LXRα with LXRE promoter DNA sequences of *Srebp1c* (P = 0.007) and *Lxr* (P = 0.009) in the presence of GW3965, as measured by ChIP (using LXRα antibody) followed by qPCR. Off-target DNA sequences ~2 kb upstream of the LXRE sites were amplified as negative controls (NC). (**D**) Vpr increased binding of LXRα to ^32^P-labeled LXRE in nuclear extracts of HepG2 cells, in the presence or absence of GW3965, as measured by gel mobility retardation assay. Data are presented as means ± SE of two replicates, and are representative of two experiments. In A and B, *P < 0.05 and **P < 0.01 compared to Vector + LXRE; ^##^ *P* < 0.01.

Liver X receptor-α (LXRα) regulates the expression of both *Srebp1c* and *Chrebp*, hence increased LXRα expression and activity could drive the upregulation of both these transcription factors and their lipogenic target genes^16,17^. To investigate whether Vpr increases expression of LXRα, we measured the ability of Vpr to activate a liver X receptor response element (LXRE)-containing promoter driving expression of firefly luciferase in two hepatocyte cell lines, HepG2 and Huh7. Vpr enhanced LXRE promoter activity in the presence of LXRα, and the effect was maximally stimulated by the LXRα agonists GW3965 and T0901317, in both cell lines (Fig. 4A,B).

We next performed chromatin immunoprecitation (ChIP) assays to quantify the effect of Vpr on expression of *Lxrα* and *Srebp1c* in HepG2 cells. Levels of *Lxrα* and *Srebp1c* promoter DNA immunoprecipitated by anti-LXRα antibody after GW3965 treatment were significantly increased in the presence of Vpr (Fig. 4C). Vpr also increased binding of LXRα to ^32^P-labeled LXRE in nuclear extracts of HepG2 cells in a gel mobility retardation assay, both in the absence and in the presence of GW3965 (Fig. 4D).

To confirm that LXRα is necessary for the effect of Vpr on the DNL pathway, LXRα knock-down HepG2 cells were created and expression of LXRα target genes measured in the presence or absence of Vpr and GW3965. mRNA levels of the LXRα target genes *Srebp1c, Chrebp* and *Lpk* were similar in HepG2 cells with or without Vpr expression, whereas overexpression of LXRα with co-expressed Vpr and addition of GW3965 led to 4.8-fold increased expression of *Srebp1c* (P = 0.006), 6-fold increased expression of *Chrebp* (P = 0.006) and a trend towards increased expression of *Lpk* (P = 0.09) (Fig. S1). Knock-down of endogenous LXRα in HepG2 cells using LxrShHepG2 resulted in significant decrease in *Srebp1c* (P = 0.005), and *Lpk* (P = 0.004) expression compared to scrambled ShHepG2 cells, despite co-expression of Vpr and addition of GW3965 (Fig. S1).

### ß-oxidation is blunted, and expression of PPARα-regulated genes of fatty acid oxidation is decreased, in liver of Vpr mice

**Figure 5:**
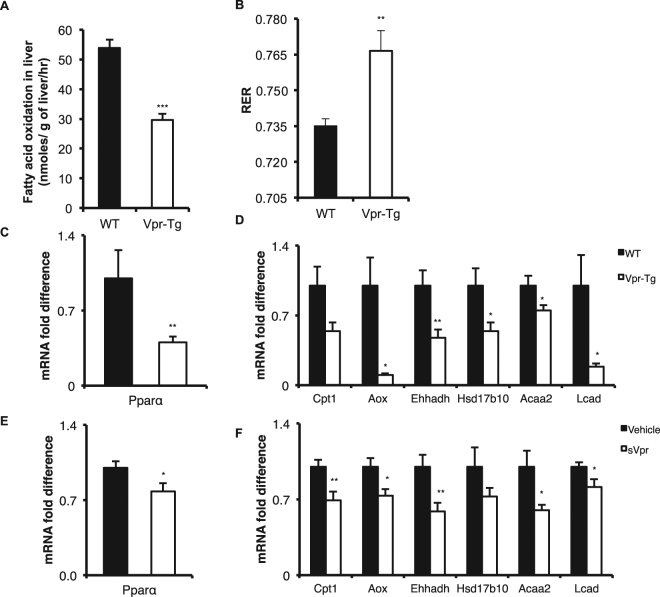
Impaired fatty acid oxidation and decreased mRNA levels of PPARα-regulated oxidative genes in liver of Vpr-Tg and sVpr-treated mice. (**A**) Fatty acid oxidation was decreased in liver of Vpr-Tg compared to WT mice (P = 0.006; N = 6 per group). (**B**) Increased respiratory exchange ratio (RER) was present during the initial 4 h of fasting in Vpr-Tg compared to WT mice (N = 5–6 per group). (**C**) *Pparα* mRNA level was decreased in liver of Vpr-Tg compared to WT mice (N = 5–6 per group). (**D**) mRNA levels of *Aox, Ehhadh*, *Hsd17b10, Acaa2* and *Lcad* were decreased in liver of Vpr-Tg compared to WT mice (N = 5–8 per group). (**E**) mRNA level of *Pparα* was decreased in sVpr-treated compared to vehicle-treated mice (N = 7 per group). (**F**) mRNA levels of *Cpt1α, Aox, Ehhadh*, *Acaa2* and *Lcad* were decreased in sVpr-treated compared to vehicle-treated mice (N = 7 per group). Values are mean ± SE. *P < 0.05, **P < 0.01, ***P < 0.001.

The rate of ß-oxidation was significantly downregulated in liver of Vpr-Tg compared to WT mice (0.30 ± 0.01 vs. 0.55 ± 0.03 nmoles/gm liver/min, P <= 0.0001) (Fig. 5A). At the whole body level, the respiratory exchange ratio (RER), measured by calorimetry was modestly increased in Vpr-Tg mice (0.76 ± 0.003 vs. 0.74 ± 0.003, P < 0.0001) (Fig. 5B), indicating blunted fat oxidation and confirming our previous report^12^. Since the genes for ß-oxidation enzymes are regulated by PPARα, we quantified their mRNA expression in liver of Vpr-Tg compared to WT mice. The mRNA level of *Pparα* was decreased in Vpr-Tg mice (Fig. 5C). mRNA levels of the PPARα-regulated beta-oxidation genes acyl CoA oxidase (*Aox*), enoyl-CoA hydratase/3-hydroxyacyl CoA dehydrogenase *(Ehhadh)*, 17β-hydroxysteroid dehydrogenase 10 *(Hsd17b10)*, acetyl-CoA acyltransferase 2 *(Acaa2)* and long-chain acyl CoA dehydrogenase (*Lcad*) were significantly decreased and that of carnitine palmitoyl transferase-1α (*Cpt1α*) showed a trend (P = 0.07) towards decrease in Vpr-Tg compared to WT liver (Fig. 5D). In liver of sVpr-treated compared to vehicle-treated mice, there were significant decreases in mRNA expression of *Pparα* and of all the PPARα-regulated fat oxidation genes except *Hsd17b10* (Fig. 5E,F).

### MTP expression is diminished and VLDL-TG export is decreased in liver of Vpr-Tg mice

**Figure 6:**
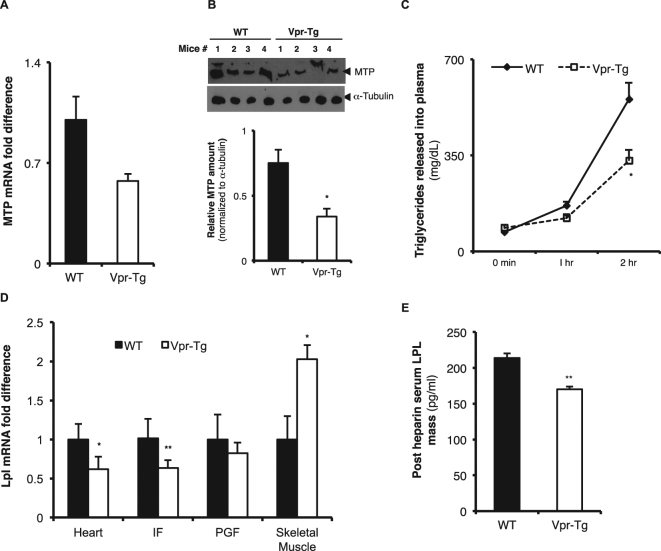
Altered levels of MTP and blunted VLDL-TG export in liver of Vpr-Tg mice. (**A**) There was a trend towards decreased *Mttp* mRNA in liver of Vpr-Tg compared to WT mice (P = 0.077, N = 5–6 per group). (**B**) Decreased protein level of MTP was present in liver of Vpr-Tg compared to WT mice (N = 4 per group). (**C**) Decreased VLDL export was observed in liver from Vpr-Tg compared to WT mice following injection of the LPL inhibitor Pluronic F-127 (N = 4 per group). (**D**) There was variably altered *Lpl* mRNA in heart, inguinal fat, perigonadal fat and skeletal muscle of Vpr-Tg compared to WT mice (N = 7–8 per group). (**E**) Decreased LPL mass was present in Vpr-Tg compared to WT mouse plasma following heparin injection (N = 4 per group). Values are mean ± SE. *P < 0.05, **P < 0.01. [Note: Immunoblot figures have been cropped to show relevant bands. Original uncropped blots are shown in Supplementary Fig. S3. No image enhancement was used, but the bluish background of the original autoradiogram was changed to gray].

Synthesis of triglycerides within hepatocytes, their packaging with apolipoprotein B and their subsequent export into the plasma are all tightly regulated by a number of exchange and transfer proteins. In regard to development of fatty liver in Vpr mice, microsomal triglyceride transfer protein (MTP) was of particular interest since PPARα enhances *Mttp* gene transcription to promote VLDL-TG export from the liver^18^, and *Mttp* knock-out induces fatty liver by inhibiting this process^19^. *Mttp* mRNA showed a trend (P = 0.07) towards diminished expression in Vpr-Tg compared to WT mouse liver (Fig. 6A), and MTP protein expression was significantly decreased in Vpr-Tg compared to WT mouse liver (Fig. 6B). The acute rise in plasma VLDL-TG levels following intraperitoneal injection of Pluronic F-127 (reflecting hepatic VLDL export) was blunted in Vpr-Tg compared to WT mice (Fig. 6C).

The finding of decreased VLDL-TG export, although consistent with the phenotype of hepatic steatosis, was surprising given the elevated plasma triglyceride levels in Vpr-Tg mice. We hypothesized that there might be a parallel defect in whole body disposal of circulating triglycerides, a process mediated in most tissues by lipoprotein lipase (LPL). Of note, others and we have shown that HIV patients have diminished whole body activity of LPL^20^. Hence we measured LPL mRNA expression in several tissues of the mice, as well as plasma LPL mass following heparin injection. Compared to WT mice, Vpr-Tg mice showed decreased LPL mRNA expression in heart and inguinal fat, whereas perigonadal fat showed no difference and skeletal muscle showed increased LPL mRNA expression (Fig. 6D). Despite these tissue variations in mRNA levels, total plasma LPL mass was significantly decreased (indicating diminished whole body LPL activity) in Vpr-Tg compared to WT mice (Fig. 6E).

### A broad array of metabolic genes in the lipogenic pathway is upregulated in Vpr-Tg liver

**Figure 7:**
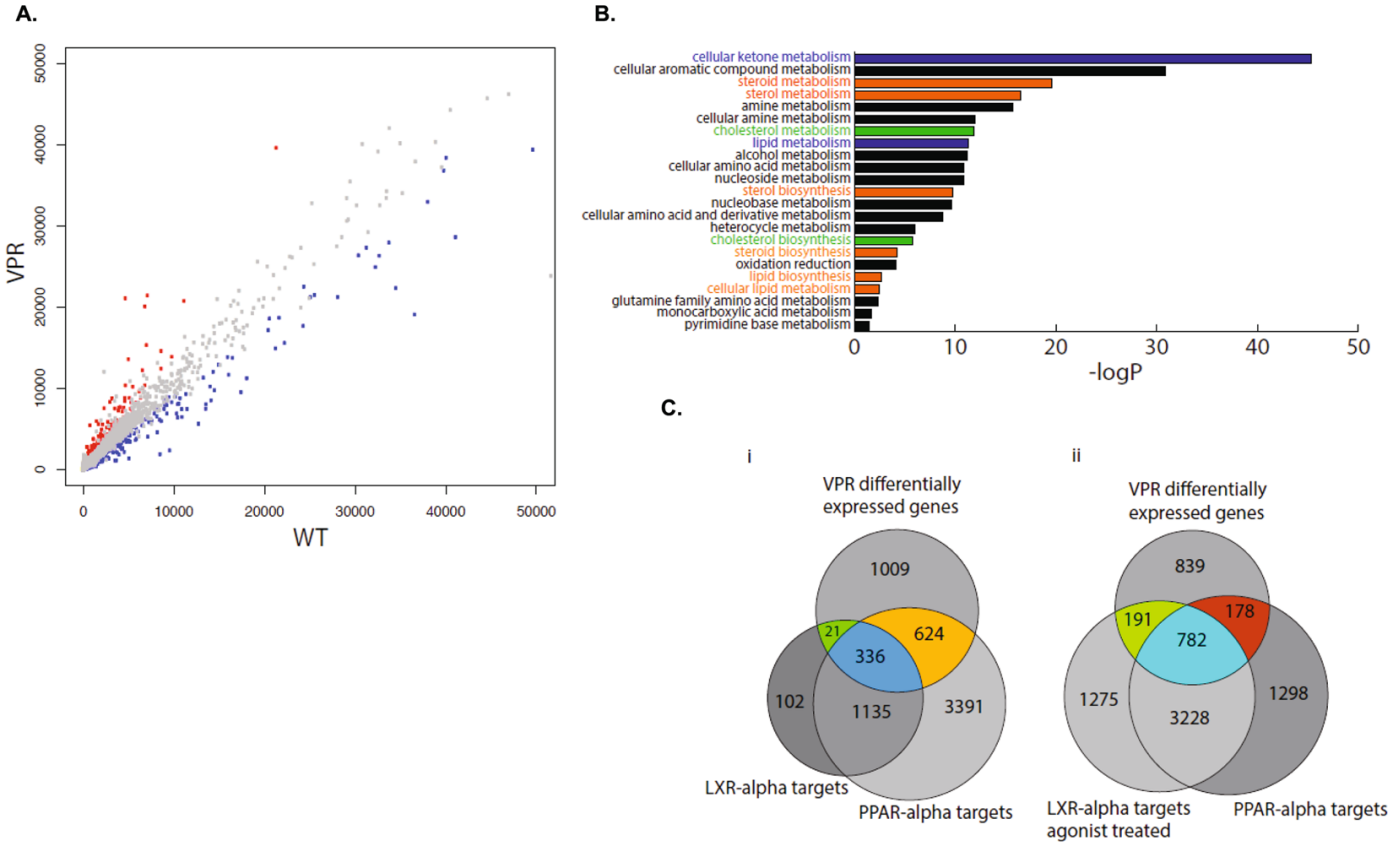
RNA-Seq analysis of liver reveals that Vpr regulates expression of a broad array of genes related to lipid metabolism. (**A**) Scatterplot of gene expression from RNA-Seq in Vpr-Tg compared with WT mouse liver. Each point represents a gene that was found to be expressed in both samples. The x-axis represents WT mouse expression and y-axis Vpr-Tg mouse expression in terms of library normalized counts. The red points are significantly upregulated genes and blue points significantly downregulated genes (P < 0.05). The plot is censored to a count of 50,000 for visualization purposes. (**B**) Gene ontology (GO) analysis for significantly different genes under the biological processes ontology framework. Of the genes for which expression differed significantly between Vpr-Tg and WT mouse livers, 46% belong to the ontology of metabolic processes. Significant ontologies within this subset are displayed (P < 0.05 using Benjamini correction, with a minimal fold change of 2). In particular, lipid and sterol metabolic pathways are highly represented. (**C**) Vpr binds preferentially to LXRα gene targets. LXRα and PPARα targets were identified by filtering a genome-wide study (27) to regions proximal to the transcriptional start site (within −10 kb to +5 kb with a 20 kb extension) using GREAT version 3.0. There are 4 datasets from fasted mice: LXRα binding sites in WT animals, LXRα binding sites in animals treated with the LXRα agonist T0901317, PPARα binding sites in WT animals, and PPARα binding sites in animals with a LXRα/β double knockout background. (i) In fasted animals, more PPARα binding sites than LXRα binding sites are recovered from the ChIP-Seq. In this state, the effect of Vpr on gene expression shows minimal overlap to LXRα binding, and is skewed towards PPARα targets. (ii) After treatment with the LXRα agonist, there is both more LXRα binding to unique regions, as well as with shared PPARα regions. Activation of LXRα by its agonist results in greater overlap with Vpr-induced gene expression.

RNA-Seq analysis demonstrated 1990 genes that were differentially expressed between WT and Vpr-Tg livers (P < 0.05). Among these, 986 genes were up-regulated and 1004 were down-regulated in Vpr-Tg liver. Figure 7A shows a scatterplot of expressed genes represented as counts in Vpr-Tg and WT livers. The relatively even distribution of significantly upregulated (red) or downregulated (blue) genes implies that Vpr is not preferentially an activator or repressor of gene expression. To futher identify the function of these gene expression changes, significant genes were evaluated by Gene Ontology (GO) for biological processes. Significantly over-represented ontologies are displayed in Fig. 7B, wherein lipid and sterol metabolism dominate the gene expression signature. This is consistent with the fatty liver phenotype of the Vpr-Tg mice and underlying molecular mechanisms.

A previous genome-wide study of LXRα and PPARα binding in mouse liver by ChIP-Seq demonstrated that they often co-bind in regulatory regions of genes associated with their classically regulated pathways and that their binding could be mutually exclusive, additive or independent in a context dependent fashion^21^. This study provided ChIP-Seq data in fasted mice for liver-specific LXRα, PPARα, LXRα in the presence of its ligand T0901317, and PPARα binding in the background of double LXRα/β knockout. We compared genes that showed significantly altered expression in Vpr-Tg mice with the available data on LXRα- and PPARα-regulated gene expression (GEO GSE35262) to determine whether Vpr-regulated gene expression correlates with LXRα binding. Fig. 7Ci shows that there are more PPARα than LXRα binding targets in mouse liver (5486 vs. 2218); however the majority of LXRα binding sites are shared with PPARα. This is consistent with data that PPARα and LXRα frequently bind at proximal albeit unique sites in the genome. In the fasted state, approximately half of the genes affected by Vpr are found in regions bound with PPARα or both PPARα and LXRα (960 sites). Upon stimulation of LXRα by its ligand T0901317 for 14d (Fig. 7Cii), the number of LXRα-bound sites increases dramatically (from 2218 without ligand to 5476 with ligand). In this condition, mimicking the post-prandial, LXR-activated state, a shift is observed towards genes bound by LXRα that are also differentially regulated by Vpr (increased from 21 to 191). This 9-fold increase in LXRα-Vpr association is not accompanied by a comparative increase in shared LXRα and PPARα sites (2.8-fold increase from 1135 to 3228), or in the overlap between the sites of all three proteins (2.3-fold increase from 336 to 782). Also, with ligand activation of LXRα, 446 sites are lost (from 624 to 178) in the overlap between Vpr and PPARα, as Vpr shifts towards co-binding with LXRα. These data show that Vpr preferentially co-regulates hepatic genes that are LXRα targets, including those that may bind to PPARα in a different context.

## Discussion

NAFLD is common in HIV-infected patients and often ascribed to ART-related hepatotoxicity or coinfection with hepatitis C or B virus, or as a manifestation of HIV/ART-associated lipodystrophy, dyslipidemia and insulin resistance^22^. However, recent studies show that HIV-1 *per se* may be an etiologic agent in HIV-associated liver disease, via binding of HIV gp120 to hepatocytes, stellate cells and Kupffer cells, or possibly by direct infection of stellate cells^23^ or hepatocytes^24^. Moreover, serum transaminase levels are frequently elevated in HIV patients without coinfections or treatment with ART^25^. Recent reports have revealed high rates of NAFLD in HIV-1 mono-infected patients (i.e., those without concomitant Hepatitis B or C infection)^2,3^. The results of the present study indicate an independent pathogenic role for Vpr in the development of HIV-associated fatty liver disease.

Four pathways of hepatic lipid metabolism are dysregulated in the Vpr mouse models, leading to steatosis: a) accelerated fatty acid flux to the liver due to lipolysis induced by Vpr-mediated coactivation of GR and corepression of PPARγ in adipose tissue^12^; b) increased hepatic DNL due to Vpr-mediated coactivation of LXRα; c) diminished hepatic fatty acid oxidation due to Vpr-mediated corepression of PPARα; d) blunted hepatic VLDL-TG export associated with decreased expression of MTP due to Vpr-mediated corepression of PPARα or accumulation of long-chain fatty acids (derived from DNL) within microsomes^18,19^. The relative contribution of each of these mechanisms to the development of fatty liver cannot be determined from the design of the current experiments, but dysregulated DNL has possibly the greatest impact, given its demonstrated role as a major contributor to NAFLD in humans^26^.

LXRα is a key regulator of fatty acid metabolism in the liver. LXRα−/− mice demonstrate deficient expression of SREBP1c and the critical lipogenic enzymes FAS, SCD-1 and ACC^27^. Liver triglycerides are significantly reduced in LXRα−/−β−/− mice^28^. Treatment with the LXR-agonist T0901317 leads to development of hepatic steatosis in WT mice^29^ via upregulated lipogenesis. Activation of the LXRα pathway has been described also in non-genetic animal models of NAFLD, such as those associated with feeding a high fat or choline deficient diet^30^. While several lipogenic genes were upregulated in Vpr mice, the greatest increase was in expression of *Scd1*. *Scd1* is regulated predominantly by the LXR-RXR heterodimer, whereas the other lipogenic genes are regulated by multiple factors^31^.

Vpr mice also demonstrated increased expression of ChREBP, which acts synergistically with SREBP1c to activate lipogenic genes. ChREBP requires cellular glucose influx and LXRα for activation^16^, whereas SREBP1c is activated by insulin and LXRα^17,32,33^. Hence, in these studies of fasting mice, it is likely that ChREBP-mediated and SREBP1c-mediated lipogenesis was occurring at a low level in the WT mice. Vpr induced a non-physiologic upregulation of both ChREBP and SREBP1c, leading to persistently elevated DNL even under fasted conditions.

Hepatic steatosis could be circumvented in the face of accelerated DNL if there were a corresponding increase in disposal of the fatty acids by mitochondrial ß-oxidation. Oxidative disposal, activated by PPARα in the absence of insulin in the fasted state, is dependent upon entry of acylated fatty acids into mitochondria by activation of CPT1, followed by sequential activation of ß-oxidation enzymes. This process is interdicted by Vpr; β-oxidation was blunted in Vpr-Tg liver due to Vpr-mediated repression of PPARα and its gene targets *Aox*, *Lcad*, *Ehhadh, Hsd10* and *Acaa2*.

Hepatic VLDL-TG export was slower in Vpr-Tg mice than in WT littermates. VLDL-TG assembly is regulated within the endoplasmic reticulum lumen of hepatocytes by MTP; this is a critical step in the export of hepatic triglycerides. *Mttp* mRNA levels were lower in liver of Vpr-Tg compared to WT littermates. This is likely due to PPARα co-repression by Vpr, as decreased PPARα levels and activity would diminish activation of the DR1 element in the *Mttp* promoter, leading to decreased mRNA expression of *Mttp* ^18^. Interestingly, decreased *Mttp* mRNA could also result from Vpr-mediated co-activation of LXRα, as increased intranuclear SREBP1c inhibits *Mttp* transcription^34^. MTP has a long half-life (T1/2 = 70 h (58, 87–88), hence it is also likely that the protein is rapidly degraded in proteasomes of Vpr-Tg hepatocytes^35^. Thus Vpr induces processes that resemble the pathogenesis of hepatic steatosis in abetalipoproteinemia, a syndrome caused by heterozygous loss-of-function mutation of the MTP gene^36^.

Despite blunted VLDL-TG export, fasting plasma triglyceride levels were elevated in the Vpr mice. A likely mechanism, given the ability of Vpr to co-repress PPARγ^12^ (a key stimulator of adipocyte lipoprotein lipase (LPL) production^37^) would be defective disposal of the exported triglycerides by LPL in adipose tissues and muscle. Indeed, Vpr-Tg mice had lower whole body abundance of LPL. Of note, hypertriglyceridemic HIV-positive humans have defective heparin-releasable plasma LPL activity, and we have shown that the “trapping” function of LPL is defective in HIV patients on ART^38^.

RNA-Seq analysis of Vpr-Tg compared to WT mouse liver revealed widespread shunting of metabolic gene regulation towards lipid metabolism in the Vpr-Tg mice. The high levels of mRNA’s directed towards lipid biosynthesis are consistent with increased DNL in the Vpr mice and indicate that Vpr activates a large number of metabolic genes within this pathway. Pathways of sterol metabolism were also markedly upregulated in Vpr-Tg liver, an important topic for future study, since HIV patients manifest defects in cholesterol metabolism^38–40^.

In the present study of young adult mice, the amount of liver fat in Vpr mice was modestly increased in the absence of a high-fat diet or treatment with ART drugs. An emerging theme in HIV medicine is that the viral or immune mechanisms “prepare the soil” for metabolic defects, while the severity of the manifestations are modulated by secondary effects of ART, diet and environmental factors. In HIV patients, Vpr-mediated NAFLD could be exacerbated by high fat consumption, hepatitis C coinfection, alcohol consumption, and the effects of ART drugs.

It is also interesting that despite a four-fold increase in triglyceride content and six-fold increase in Oil Red O stained area in liver of Vpr mice, histology revealed a predominant increase in microsteatosis with some macrosteatosis, and AST rather than ALT was elevated. This pattern resembles liver injury associated with mitochondrial dysfunction, as in Reye’s syndrome, acute fatty liver of pregnancy and valproate-associated fatty liver disease^41–43^. Vpr is known to disrupt mitochondrial function via increased permeability of the mitochondrial outer membrane^44^ and apoptosis^45^. Thus hepatic steatosis caused by HIV-1 could have distinctive features such as mixed macro- and microsteatosis, as noted in a systematic study correlating clinical and biochemical characteristics with histology of liver biopsies from mono-infected HIV patients^6^. The present data show that the virus, via Vpr and other mechanisms, could contribute to a range of metabolic abnormalities previously described in HIV patients, but lacking a clear etiology. However, these investigations do not address issues that should inform future translational studies: interactions of currently used ART drugs or high fat/high calorie diets with Vpr, liver effects of intact HIV, modulation by other metabolically significant HIV proteins such as Nef or Tat, and direct (biopsy) evidence for the association of Vpr in the liver with coexisting NAFLD in HIV patients.

Serum lipid and liver function test values as well as SREBP1c mRNA and protein levels were higher in the controls of the Vpr-Tg versus WT mouse experiments than in the controls of the sVpr-treated versus vehicle-treated mouse experiments. Dietary differences in the two sets of experiments could explain some of these variances - in the Vpr-Tg/WT studies, the mice were fed a high-protein diet for 3 wks (to induce transgenic protein expression in the Vpr-Tg mice), whereas in the sVpr-treated/vehicle-treated studies, all mice received regular chow. Mice on high protein diets have been shown to have elevated plasma triglyceride levels compared to those receiving a normal protein diet^46^. Long-term amino acid feeding also activates the hepatic mTORC1 pathway both *in vivo* and *in vitro*, leading to increased SREBP1c1c expression in mice^47^. The FVBN mice used in the two sets of experiments were derived from different breeding colonies, and it is not uncommon for animals of the same parent strain bred under different conditions to have variable normal ranges for a number of metabolites (including transaminase levels).

We propose a model wherein HIV-1 Vpr, either produced within hepatocytes or taken up into liver cells may (following release of Vpr into extracellular spaces from HIV replicating in immune cells within reservoirs), dysregulates several pathways of hepatic lipid metabolism, including those of lipogenesis, fatty acid oxidation and VLDL-TG export. It is likely that Vpr’s effects on other tissues, such as hyperlipolysis in adipose depots, contribute to hepatic steatosis. The regulation by Vpr of a large number of hepatic genes related to lipid and sterol metabolism suggests that Vpr may have a role in additional metabolic abnormalities that develop with HIV infection. A caveat to these proposals is that the current data are derived from mouse studies and that their importance to NAFLD in HIV-infected humans remains to be determined. Nevertheless, taken together with our previous finding that virus-free Vpr circulates in the blood of HIV patients on ART with undetectable plasma viral load^12^, these data support a potential direct role for an HIV factor in the pathogenesis of NAFLD.

## Materials and Methods

### Study design

We utilized Vpr-Tg and pharmacologic (synthetic Vpr-treated) mouse models to test the hypothesis that Vpr interferes with key transcription factors to induce hepatic steatosis. For gene and protein expression experiments, groups of 5 to 7 Vpr mice (Vpr-Tg or sVpr-treated WT mice) were compared to similar numbers of control mice (WT littermates or vehicle-treated WT mice); sample sizes were based on our previous data regarding the effects of Vpr on adipose tissues^12,48^. For histological, biochemical or serologic experiments, samples sizes were smaller or larger, as appropriate. Male 14-week-old FVBN mice were used in most experiments, with 28-week old mice used in some experiments to demonstrate persistence of the phenotype with aging.

### Vpr-Tg mice

Protocols were approved by the Baylor IACUC. All animals received humane care according to criteria in the “Guide for the Care and Use of Laboratory Animals” (NIH publication 86–23 revised 1985). FVBN mice expressing PEPCK promoter-driven Vpr under control of a tetracycline-repressible (tTA) system were constructed at NIH^48^. Two transgenic lines were re-derived at Baylor. We reported previously that these mice express Vpr in liver, adipose tissues and kidneys, and secrete Vpr into the plasma where it can be measured by immunoaffinity capillary electrophoresis^48^. Plasma Vpr levels in Vpr-Tg mice (98.7–329.4 pg/ml) are within the range of Vpr levels measured in plasma of HIV-infected patients receiving ART, with or without detectable plasma viral load (0.32–435.61 pg/ml)^12^.

### Synthetic Vpr (sVpr)

was produced by solid-state peptide synthesis, purified, characterized by sequencing and mass spectrometry, and compared to viral Vpr by SDS-PAGE and immunoblot^49^. Stability of the peptide in aqueous solution was confirmed by dynamic light scattering, circular dichroism and ^1^H-NMR spectroscopy^49^.

### Continuous delivery of sVpr

Alzet pumps, model 1002 (Durect, Cupertino, CA) containing aqueous solution of sVpr or sterile water, were implanted subcutaneously in WT mice with a delivery rate of 0.25 μL/h to administer 5 μg of sVpr/24 h for 14d. Subcutaneous delivery of sVpr results in sustained, high Vpr concentrations in the plasma (median 839.2 pg/ml, range 381.9–983.3 pg/ml)^12^.

### Measuring effects of Vpr on mouse liver, *in vivo* and *ex vivo*

For experiments utilizing the transgenic model, Vpr-Tg and WT littermates were placed on high protein diet (Harlan-Teklad, Indianapolis, IN) for three weeks (to activate the PEPCK/tTA promoter) prior to measurements or sacrifice^12,48^. For experiments utilizing the pharmacologic model, sVpr or vehicle was infused continuously via subcutaneous Alzet pump into WT mice for two weeks prior to measurements or sacrifice^12^. For terminal experiments, mice were fasted for 15 h and euthanized in the morning. Blood and tissues were collected and plasma immediately separated. Tissues were snap-frozen for RNA and protein extraction and stored at −80 °C, or fixed in 10% formalin for immunohistochemistry.

#### Liver function tests

Plasma levels of total bilirubin, triglycerides, AST, ALT and alkaline phosphatase were measured using standard commercial assay kits.

#### Quantification of hepatic lipids

*A. Thin layer chromatography (TLC):* Lipids were extracted from 0.2 g liver, dried under nitrogen, reconstituted in chloroform and loaded onto a TLC plate with standards to identify lipid species. Lipid fractions were visualized using iodine vapor. TLC plates were scanned and lipid species quantified by densitometry using Image J software. *B. Colorimetric method:* Frozen liver tissue (~100 mg) was homogenized in 1.6 ml PBS and protein concentration determined using D_C_ Protein Assay (Bio-Rad, Hercules, CA). Lipid was extracted using chloroform:methanol (2:1) and 0.1% sulfuric acid as described^50^. An aliquot of the organic phase was collected, dried with chloroform containing 1% Triton, and resuspended in water. Triglyceride content was determined using kits (Wako Chemicals) in microtiter plates and normalized to protein concentration of the homogenate.

#### Oil Red O staining for hepatic fat

Cryostat sections of liver were cut, fixed in 10% formalin and stained with Oil Red O; 3–4 pictures of different fields were taken at 20X magnification. Staining was quantified using Image J software.

#### Histology

Livers were removed, fixed with 4% paraformaldehyde in PBS for 24 h at 4 °C, paraffin-embedded and cut at 4 μm thickness. Sections were stained with hematoxylin-eosin and examined by two independent investigators masked to the treatment groups. Microvesicular vs. macrovesicular steatosis, ballooning degeneration and lobular inflammation were evaluated as previously described^30^.

#### mRNA levels

Total RNA was extracted from liver using Trizol (Invitrogen, Grand Island, NY), transcribed using the RNA-to-cDNA kit (Applied Biosystems, Grand Island, NY), and PCR performed using the TaqMan assay. PPARα and LXRα target genes included: *Pparα Cpt1α Aox*, *Lcad*, *Ehhadh, Hsd10* and *Acaa2*, *Mttp*, *Chrebp*, *Srebp1c, Lpk*, *Dgat*, *Fasn, Scd1* and *Acc*. *Pgk1* mRNA was used for normalization.

#### Immunoblotting

Total protein was extracted from mouse liver using RIPA buffer with phosphatase and protease inhibitors. Nuclear extracts were prepared from liver tissue. Protein was quantified using the Bradford reagent, resolved by SDS-PAGE, transferred to nitrocellulose membranes and identified by enhanced chemiluminescence (Thermo Fisher, Grand Island, NY). Multiple replicate blots were prepared for each experiment, each with 50 µg total protein loaded per lane, and each probed with a different antibody. Primary antibodies were obtained from Cell Signaling (Danvers, MA: FASN, ACC, Ser79-phospho-ACC and α-tubulin), Abcam (Cambrdge, MA: SREBP1c and ChREBP), Santa Cruz (Dallas, TX: MTP), Millipore (Billerica, MA: TBP), and Sigma (St. Louis, MO: β-actin). Immunoblots were scanned and densitometry quantified using Image J software.

*Hepatic DNL* in fresh liver tissue was measured by incorporation of ^14^C acetate into lipids, as previously described^51–54^. Mouse primary hepatocytes were prepared and cultured overnight in DMEM, 10% FBS (HyClone), 100 nM insulin (Novo Nordisk, Plainsboro, NJ), and 100 nM dexamethasone (Sigma, St. Louis, MO). The hepatocytes were incubated in culture medium containing 74 KBq/ml [2–14C] sodium acetate (2.07 GBq/mmol; Perkin Elmer, Waltham, MA) for 1 h, then lysed with 1 N NaOH solution. Sterols were separated using petroleum ether. The aqueous layer was acidified with 9 M sulfuric acid and lipids extracted with petroleum ether. Radioactivity in the lipid fraction was measured using a ß-scintillation counter.

*Hepatic β-oxidation* was measured as previously described^55^. In brief, liver homogenate was prepared from fresh tissue in ice-cold buffer using a Dounce homogenizer and filtered through a 70 µm nylon membrane. The homogenate was placed in incubation buffer containing carnitine, NAD, ATP, cytochrome C, MgCl_2_, coenzyme-A and ^14^C-palmitate complexed with fatty acid-free BSA for 1 h. The CO_2_ released was trapped by hyamine hydroxide, and ^14^CO_2_ radioactivity measured using a ß-scintillation counter.

#### Hepatic VLDL export

Mice were fasted for 4 h, then injected intraperitoneally with the copolymer surfactant and lipoprotein lipase inhibitor Pluronic F-127 (Sigma-Aldrich, St. Louis, MO) at a dose of 2 mg/g body weight, to quantify VLDL particle secretion from the liver. Blood was collected from the tail vein at 0, 60 and 120 min to measure plasma triglyceride levels.

#### LPL release assay

Mice were fasted for 4 h. Heparin was injected intraperitoneally (50 U/kg body weight) and blood collected 15 min later. Plasma was separated and stored at −80 °C until use. Lipoprotein lipase (LPL) mass was measured by ELISA (LSBio Inc., Seattle, WA).

#### Calorimetry

Whole body calorimetry (Oxymax, Columbus, OH) was performed on Vpr-Tg and WT mice to measure O_2_ consumption (VO_2_) and CO_2_ production (VCO_2_) for 5 h following onset of fasting. Respiratory exchange ratio (RER = VO_2_/VCO_2_) was calculated to provide an index of the fuel substrate being oxidized.

### Measuring effects of Vpr on hepatocytes and liver *in vitro* and *ex vivo*

#### Transfection and luciferase assay

HepG2 or Huh7 cells were cultured in DMEM 10% FBS at 37 °C. Before transfection, the cells were plated at ~70% confluency in 6-well plates; on the following day, they were transiently transfected with a LXRE-luciferase reporter construct (a gift of Dr. D. Moore, Baylor College of Medicine) with empty vector, Vpr, LXRα (from Dr. D. Moore) or Vpr + LXRα using the PolyJet reagent (SignaGen, Rockville, MD). The culture media was changed 12 h after transfection, with or without addition of LXRα agonist GW3965 or T0901317 (Millipore, Billerica, MA). On the next day, the cells were washed with chilled PBS, followed by lysis with 1X PLB buffer (Promega, Madison, WI). Luciferase activity was measured using a kit (Promega, Madison, WI).

#### Gel mobility shift assay

HepG2 cells were grown in 100 mm culture plates until 70% confluent. Transient transfection of LXRα or empty vector was performed using PolyJet^TM^. After 12 h, the culture medium was changed with or without addition of GW3965. On the following day, nuclear extracts were prepared from the transfected cells. For the electrophoretic mobility assay, 10 μg nuclear extracts were mixed with 16 fmoles (20,000 cpm) of ^32^P-labeled LXRE probe (Supplementary Table S1) for 15 min at 37 °C in a reaction volume of 16 μL. The reaction mix consisted of 2 µg of Poly dI:dC in a binding buffer (25 mM HEPES, pH 7.9, 0.5 mM EDTA, 0.5 mM, DTT, 1% Nonidet P-40, 5% glycerol, 50 mM Nacl) with or without sVpr. The DNA-protein complex was resolved by electrophoresis in 5% native polyacrylamide gel at 150 V for 3 h in buffer containing 50 mM Tris, 200 mM glycine (pH 8.5), 1 mM EDTA. The gel was dried and exposed to X-ray film.

#### Chromatin immunoprecipitation assay

ChIP was performed as previously described (http://www.abcam.com/ps/pdf/protocols/x_CHip_protocol.pdf). Briefly, HepG2 cells were grown in 100 mm culture plates. The cells were transiently transfected with empty vector, Vpr, LXRα or Vpr + LXRα, together with PolyJet. The culture medium was changed after 12 h, with or without addition of GW3965. Transfected cells were fixed with 1% formaldehyde at RT for 15 min, then re-suspended in lysis buffer and sonicated, resulting in DNA fragments of 200–1,000 bp. Immunoprecipitation was performed using anti-LXRα (Sigma Aldrich, St. Louis, MO) or anti-rabbit isotype antibody (Abcam, Cambridge. MA) as control for nonspecific binding, and protein A/G agarose beads (Santa Cruz, Dallas, TX). The reactions were incubated overnight at 4 °C on a rotator. Protein-bound, immunoprecipitated DNA was eluted by adding elution buffer and incubating at 65 °C for 4 h, followed by purification using a PCR purification kit (Qiagen, Valencia, CA). PCR was performed to quantify DNA at specific loci using a set of primers that target the LXRE promoter sequence (Supplementary Table S2). As a negative control, non-targeting sequence primers were designed using UCSC hg19 Genome Build by seeking locations within the genome that would result in amplicon GC content and length approximating those sequences corresponding to target primer pairs (Supplementary Table S2).

#### LXR Knockdown in HepG2 cells

LXRShRNA and scrambled ShRNA were purchased from OriGene (Rockland, MD). 1.5 × 10^6^ HepG2 cells were plated in a 6-well plate and infected the following day with lentiviral particles containing LXRShRNA or scrambled RNA (mixed with polybrene). The culture medium was changed after 12 h, followed by reinfection. After 18 h, the cells were grown in a 100 mm plate. Stable cell lines were created using puromycin selection (5 ug/ml initially, decreased over time to 0.5 ug/mL). Positively selected LXR k/d HepG2 cells were maintained in culture medium with 0.5 ug/ml puromycin.

Positively selected LXRShHepG2 cells, scrambled ShHepG2 cells or uninfected HepG2 cells were plated in 6-well plates and transiently transfected with empty vector, Vpr or Vpr + LXRα, using Lipofectamine 2000 (Invitrogen, Grand Island, NY). The culture medium was changed after 12 h, with addition of the LXRα ligand GW3965. On the following day, RNA was extracted using Trizol (Invitrogen), transcribed using the RNA-to-cDNA kit (Applied Biosystems, Grand Island, NY), and PCR performed using the TaqMan assay for *Chrebp*, *Srebp1c*, *Lpk* and *Gapdh*.

#### RNA-Seq protocol and data analysis

RNA was extracted from liver tissue and its quality checked using a NanoDrop spectrophotometer and Agilent 2100 Bioanalyzer. The Illumina TruSeq RNA library preparation protocol was followed. A double-stranded DNA library was created using 250 ng total RNA, quantitated by picogreen, and fragments prepared for hybridization onto a flowcell. ERCC RNA controls were first spiked into each sample, then cDNA was created using the fragmented 3′-polyA selected portion of total RNA and random primers. Libraries were created from cDNA by blunt-ending the fragments, attaching an adenosine to the 3′-end, and finally ligating unique adapters to the ends. The ligated products were amplified with 15 cycles of PCR. The resulting libraries were quantitated by the NanoDrop spectrophotometer and fragment size assessed with the Agilent 2100 Bioanalyzer. Quantitative PCR assay was performed on the libraries to determine concentration of adapter ligated fragments using the Applied Biosystems ViiA 7 Quantitative PCR instrument and a KAPA Library Quant Kit. Samples were pooled equimolar, re-quantitated by qPCR, and re-assessed on the Bioanalyzer. Using the pooled concentration from qPCR, the library pool was loaded onto one lane of a high output mode flow cell at a concentration of 18 pM for cluster generation on the cBot and sequencing on HiSeq. 2500, at a read length of 100 bp, paired-end.

The raw sequence reads were 101 nucleotides. To increase mappability, low-quality nucleotides were trimmed from the 5′-ends of the reads. The resulting 90-nucleotide pair-ended reads were mapped to the mouse genome (mm10) using STAR^56^ with NCBI RefSeq genes as reference. To reduce possible PCR biases, read duplicates were removed using Picard tools (http://broadinstitute.github.io/picard/). HTseq (http://www-huber.embl.de/users/anders/HTSeq) was used to determine the number of reads falling in known genes. DESeq. 2^57^ was used to to detect genes differentially expressed between control and Vpr livers, and P < 0.05 was considered statistically significant. All genes with a library normalized expression count of at least 10 were plotted and significant genes were highlighted using R. The significant genes were analyzed for enriched gene ontologies using DAVID, limiting to biological processes. Finally, LXR and PPARα ChIP-Seq data downloaded as called peaks from GEO GSE35262 were compared to gene expression in the present study using GREAT and assigning peaks to genes from −10kb to +5 kb of known TSS with a 20 kb extension. Venn diagrams were constructed after intersections were identified using custom awk scripts.

### Statistics

All experiments involved two-way comparisons between Vpr and WT groups. Data sets were checked for normality using the Shapiro Wilk test. Two-tailed, unpaired t-tests for unequal variance were used if data were normally distributed for both groups. The Wilcoxon rank sum test was used if data were not normally distributed for either group. P < 0.05 was considered significant. Data from *in vitro* experiments of promoter activity (luciferase assays or ChIP) were compared using two-tailed, unpaired t-tests with Bonferroni-Dunn correction for multiple comparisons.

### Data availability

The datasets generated or analysed during the current study are available from the corresponding author on reasonable request.

## Electronic supplementary material

HIV-1 viral protein R (Vpr) induces fatty liver in mice via LXRα and PPARα dysregulation: implications for HIV-specific pathogenesis of NAFLD
