# Supplementary material for: HIV-1 viral protein R (Vpr) induces fatty liver in mice via LXRα and PPARα dysregulation: implications for HIV-specific pathogenesis of NAFLD
Source: Sci Rep. 2017 Oct 17;7:13362. doi: 10.1038/s41598-017-13835-w (PMC5645472; doi:10.1038/s41598-017-13835-w)
Supplement: Supplementary file 1 — HIV-1 viral protein R (Vpr) induces fatty liver in mice via LXRα and PPARα dysregulation: implications for HIV-specific pathogenesis of NAFLD [file 41598_2017_13835_MOESM1_ESM.doc]

**HIV-1 viral protein R (Vpr) induces fatty liver in mice via LXRα and PPARα dysregulation: implications for HIV-specific pathogenesis of NAFLD**

Neeti Agarwal^1^, Dinakar Iyer^1^†, Chiara Gabbi^2,#^, Pradip Saha^1^, Sanjeet G. Patel^3^, Qianxing Mo^4^,

Benny Chang^1^, Biman Goswami^1^, Ulrich Schubert^5^, Jeffrey B. Kopp^6^, Dorothy E. Lewis^7^,

Ashok Balasubramanyam^1,8*^

**Supplementary Information:** Figures: 4; Tables: 2


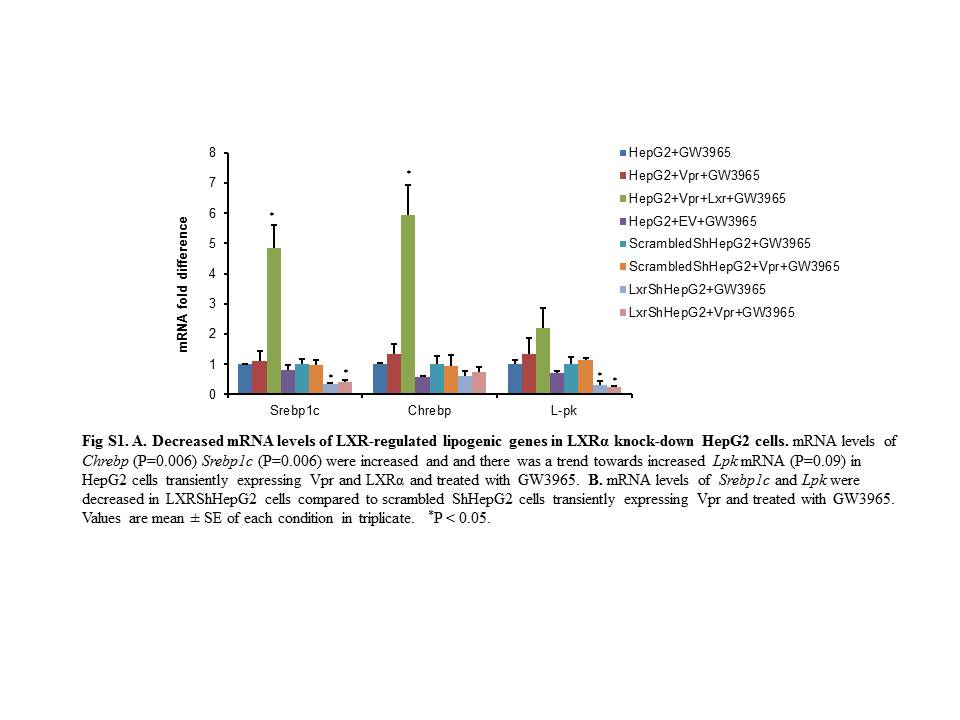


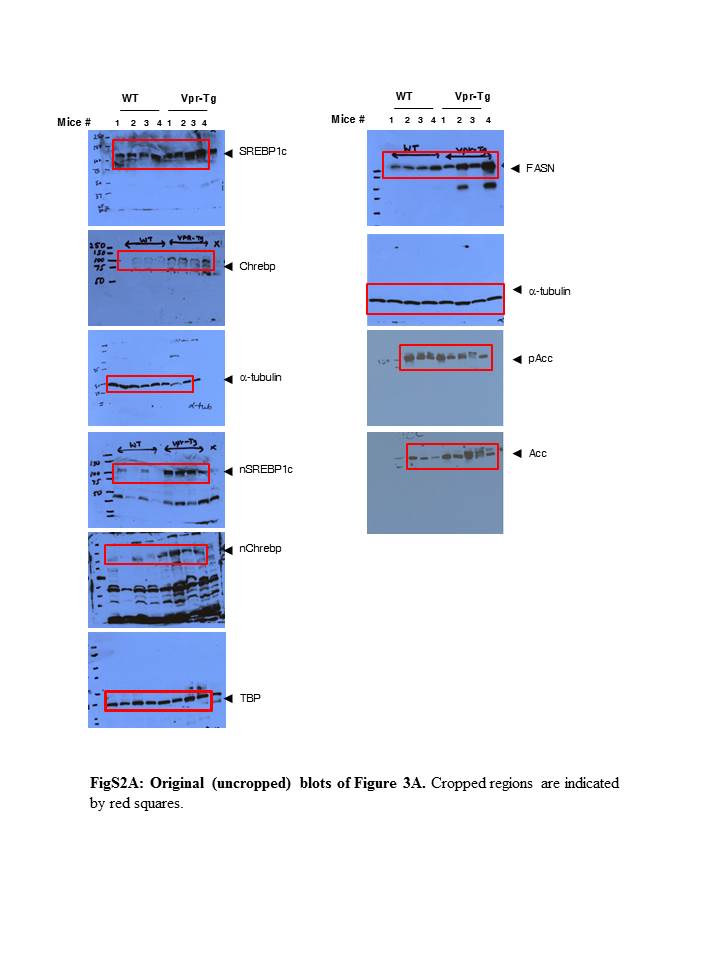


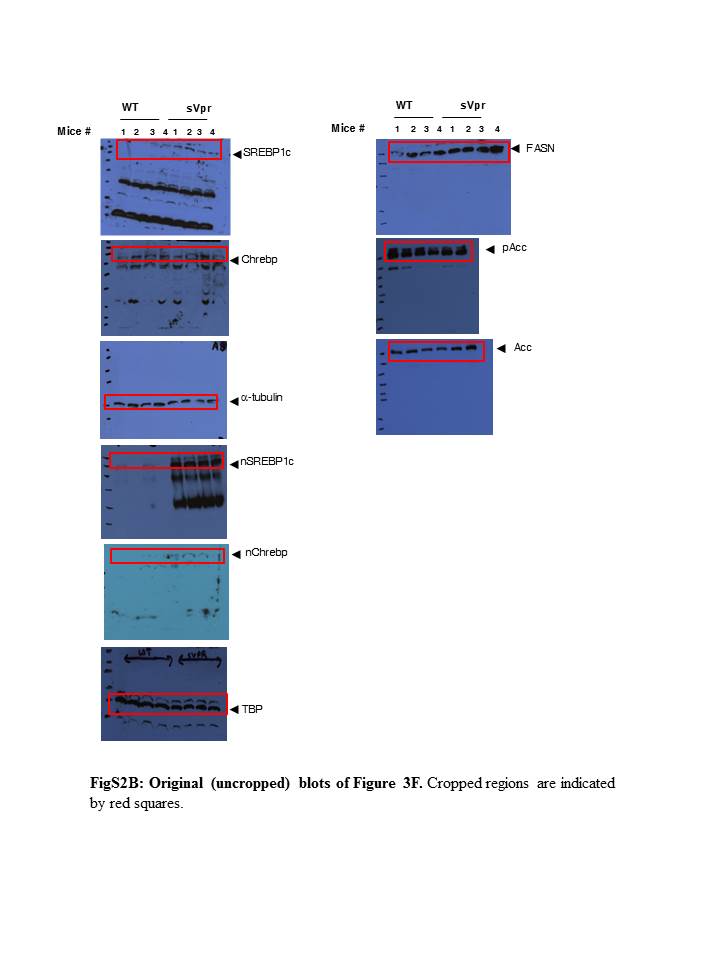


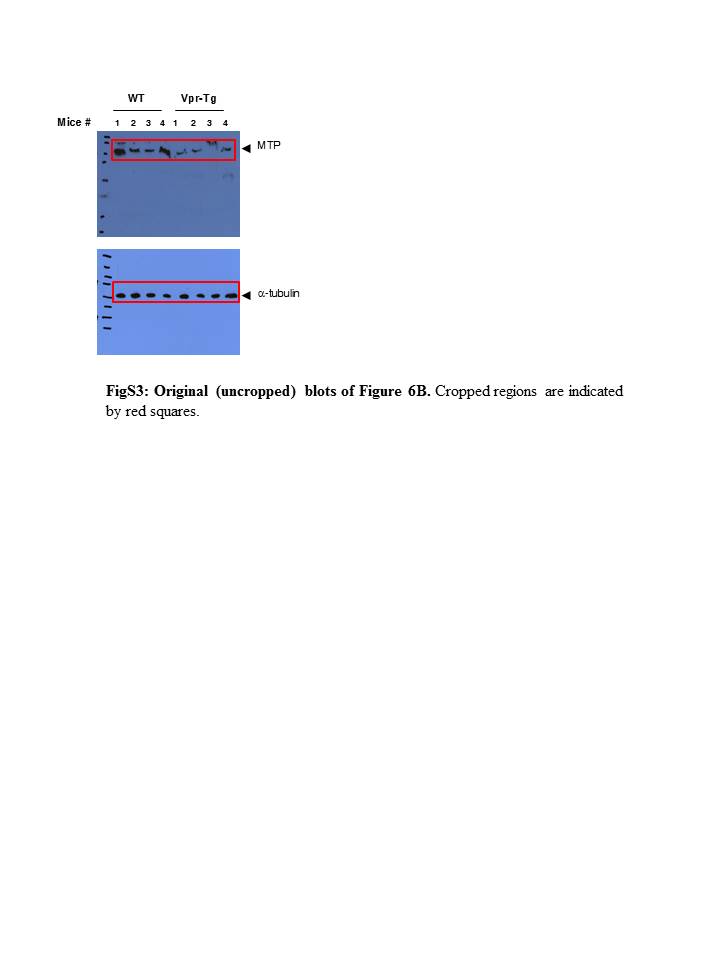


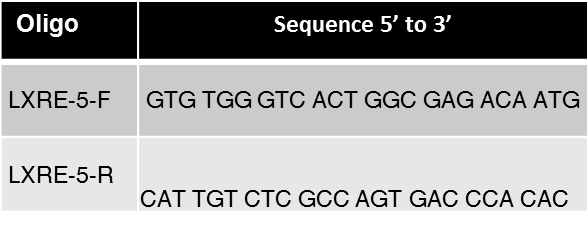

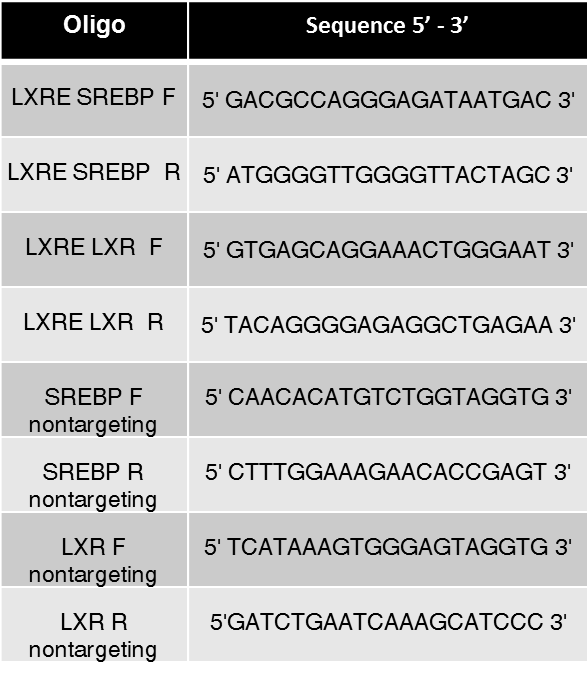


**Supplementary Table S1. LXRE probe sequences for EMSA**

**Supplementary Table S2. Primer sequences for ChIP qPCR**
